# The multifunctional RNA-binding protein Staufen1: an emerging regulator of oncogenesis through its various roles in key cellular events

**DOI:** 10.1007/s00018-021-03965-w

**Published:** 2021-10-11

**Authors:** Shekoufeh Almasi, Bernard J. Jasmin

**Affiliations:** 1 Department of Cellular and Molecular Medicine, Faculty of Medicine, University of Ottawa, Ottawa, ON K1H 8M5 Canada; 2 The Eric J. Poulin Centre for Neuromuscular Diseases, Faculty of Medicine, University of Ottawa, Ottawa, K1H 8M5 Canada

**Keywords:** Staufen1, Cancer, RNA metabolism, Cell functions

## Abstract

The double-stranded multifunctional RNA-binding protein (dsRBP) Staufen was initially discovered in insects as a regulator of mRNA localization. Later, its mammalian orthologs have been described in different organisms, including humans. Two human orthologues of Staufen, named Staufen1 (STAU1) and Staufen2 (STAU2), share some structural and functional similarities. However, given their different spatio-temporal expression patterns, each of these orthologues plays distinct roles in cells. In the current review, we focus on the role of STAU1 in cell functions and cancer development. Since its discovery, STAU1 has mostly been studied for its involvement in various aspects of RNA metabolism. Given the pivotal role of RNA metabolism within cells, recent studies have explored the mechanistic impact of STAU1 in a wide variety of cell functions ranging from cell growth to cell death, as well as in various disease states. In particular, there has been increasing attention on the role of STAU1 in neuromuscular disorders, neurodegeneration, and cancer. Here, we provide an overview of the current knowledge on the role of STAU1 in RNA metabolism and cell functions. We also highlight the link between STAU1-mediated control of cellular functions and cancer development, progression, and treatment. Hence, our review emphasizes the potential of STAU1 as a novel biomarker and therapeutic target for cancer diagnosis and treatment, respectively.

## Introduction

**Fig. 1:**
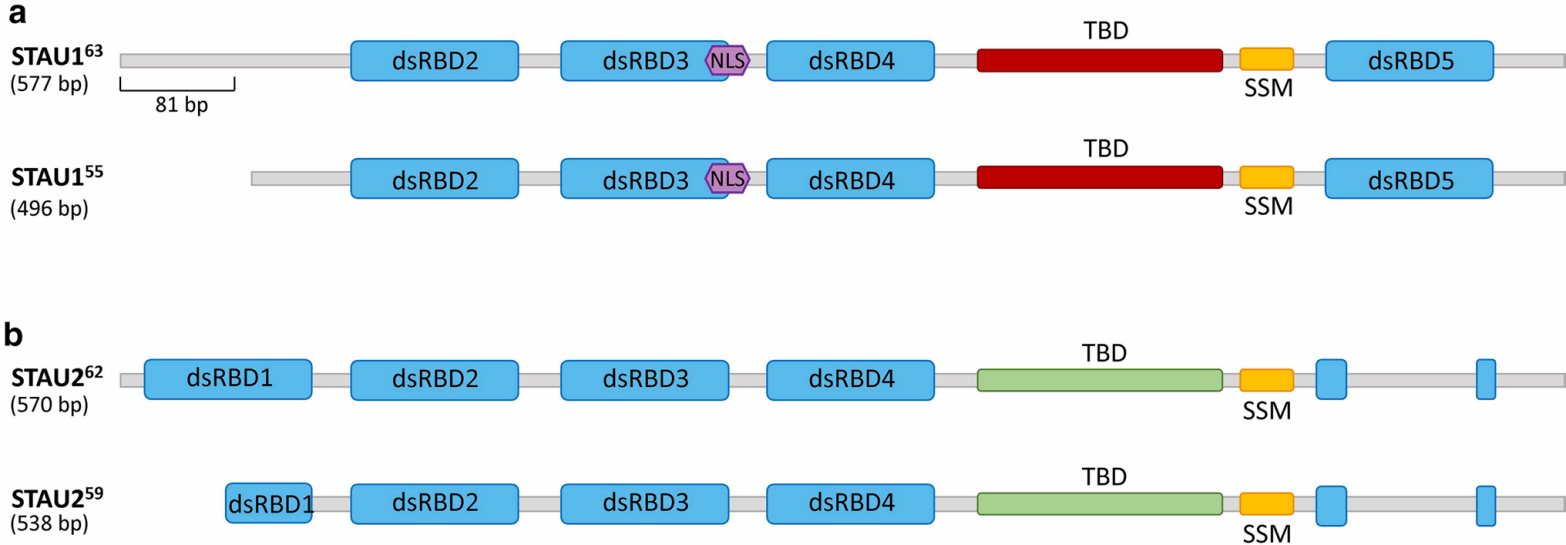
Schematic presentation of STAU1 and STAU2 isoforms. **a**, **b** All isoforms contain the double-stranded RNA-binding domains (dsRBDs) (light blue boxes), the nuclear localization signal (NLS) (purple), the tubulin binding domain (TBD) (red and green), and the reported Staufen-swapping motif (SSM) (yellow). Despite the high sequence similarity in dsRBDs of STAU1 and STAU2, the TBD motifs of two proteins exhibit great sequence variations (shown in different colors, red and green). The C-terminal end is varied among different splicing variants of each STAU protein

The double-stranded RNA-binding protein (dsRBP) Staufen was first described in Drosophila oocytes as an essential regulator of the posterior–anterior localization of mRNAs. The regulatory effect of Staufen on the distribution of maternal mRNAs is key to the early development of Drosophila embryo [1]. Later, different homologs of Staufen were identified in Caenorhabditis elegans (C.elegans) and Caenorhabditis briggsae [2]. Although a single Staufen gene has been detected in invertebrates, two independent Staufen genes have been described in vertebrates including mammals, fish, amphibians, and birds [3]. Despite the structural similarities and conserved domains among different orthologs, Staufen may exert distinct functions in each organism depending on the specific developmental stages and environmental circumstances [4]. Two mammalian orthologues of Staufen, Staufen1 (STAU1) and Staufen2 (STAU2), play distinct cellular functions (Fig. 1). Despite some similarities in their sequences and RNA-binding domains, only ~ 30% overlap has been observed among the mRNA content of their messenger ribonucleoprotein (mRNP) complexes [5]. Moreover, STAU1 is ubiquitously expressed in most cell types and tissues while STAU2 is predominantly expressed in the brain and heart [6].

In the past two decades, a growing body of literature has explored the role of STAU1 in RNA localization [7, 8], splicing [9, 10], stability [11, 12], translation [13, 14], and decay [15–17]. Findings from such studies led to additional investigations into the mechanistic roles of STAU1 in a variety of cell functions. As a result, STAU1’s involvement in cell proliferation [18, 19], differentiation [20, 21], migration [22], apoptosis [23], autophagy [24], and stress response [25–27] has been uncovered. Given this, dysregulation of STAU1’s expression and/or function has been linked with disrupted cellular functions and with the pathophysiology of several diseases including neurodegenerative [24, 27, 28] and neuromuscular disorders [26, 29, 30], as well as cancer [31–33]. In this review, we focus on the role of STAU1 in cell functions linked to cancer. More specially, we highlight the impact of STAU1 expression/dysregulation on the development and prognosis of cancers leading to the notion that STAU1 is a novel disease biomarker and therapeutic target for cancer.

### STAU1 domains, structure, and binding sites

The STAU1 protein is encoded by the human gene *STAU1* located on the long arm of chromosome 20 (20q13.13), which contains 19 exons spreading over 75.43 kb. Mature STAU1 mRNAs produce five alternative splice variants that are different in their 5′UTR regions. The two major STAU1 variants generate STAU1^55^ and STAU1^63^ proteins that contain multiple double-stranded RNA-binding domains (dsRBD), a microtubule-binding domain (TBD), and a STAU1-swapping motif (SSM) (Fig. 1) [34]. Of the four dsRBD domains, dsRBD3 and dsRBD4 are required for direct binding of STAU1 to mRNAs. The TBD domain is implicated in STAU1 binding to tubulin which facilitates RNA transport via cytoskeleton-dependent mechanisms. The dsRBD2, dsRBD5, and SSM motifs are involved in STAU1 homodimerization [16]. Through its dsRBD domains, STAU1 homodimers [35, 36] directly bind to target mRNAs thereby regulating different aspects of RNA metabolism. To date, STAU1-binding sites (SBS) have been located in the 3ʹUTR, 5ʹUTR, and coding regions of over 1000 transcripts [5, 37].

STAU1 binding sites can be classified into two main classes. The first class includes paired Alu elements in 3′ UTRs. Alu repeats are repetitive and mobile elements located in the genome of primates. Alu sequences are ∼300 nucleotides long and they are classified as short interspersed nuclear elements (SINEs). The human genome contains ~ 1 million Alu elements spread over intergenic regions, introns, and 3′ UTRs. It was previously shown that closely spaced Alu pairs can form dsRNA secondary structures which may serve as STAU1-binding sites [38]. These Alu-pair Stau1-binding sites are highly enriched in distal 3′ UTRs and in the 3′ side of intergenic regions in the immediate vicinity of the annotated 3′ UTRs. Most Alu-pair Stau1-binding sites contain multiple Alu pairs that form several helices containing over 30 base pairs interrupted by 2 to 10 nucleotide loops [38]. However, the non-target 3′ UTRs are shorter in length and separated by longer loops. Moreover, the presence of short stem-loop structures and inverted Alu pairs (referred to as Inverted Repeat Alus or *IRAlus*) separated by short loops were shown to mediate STAU1 binding to target RNA [39].

The second type of STAU1-binding site is non-Alu sequences. Non-Alu 3ʹUTR binding sites have been reported in multiple target mRNAs. For instance, complex structures consist of a few hundred nucleotides containing several STAU1 binding helices that have been observed in the 3′UTR of several targets including Arf1 [13]. Moreover, GC-rich STAU1-binding sites located in the 3′UTR or 5′UTR of target mRNAs have also been identified. These binding sites are kinetically labile, and the extent of STAU1 occupancy on these sites depends on their propensity to form secondary structure which is driven by high GC content [5]. Furthermore, it has been determined that CDS regions with high GC content also tend to form secondary structures which can serve as STAU1 binding sites. Interestingly, the efficacy of STAU1 binding to these sites is completely independent of STAU1 interactions with the 3ʹUTR of the same mRNA [12].

### The role of STAU1 in RNA metabolism

RNA metabolism refers to events involved in RNA synthesis, folding, modification, processing, translation, and decay [39]. RNA-Binding Proteins (RBPs) play crucial roles during these events via dynamic binding to pre-mRNAs and mRNAs as well as by regulating RNA processing [40, 41]. The dynamic interaction between RBPs and coding, untranslated, and non–protein-coding RNAs in ribonucleoprotein (RNP) complexes allows their stable binding throughout these events from mRNA synthesis to degradation [42].

**Fig. 2:**
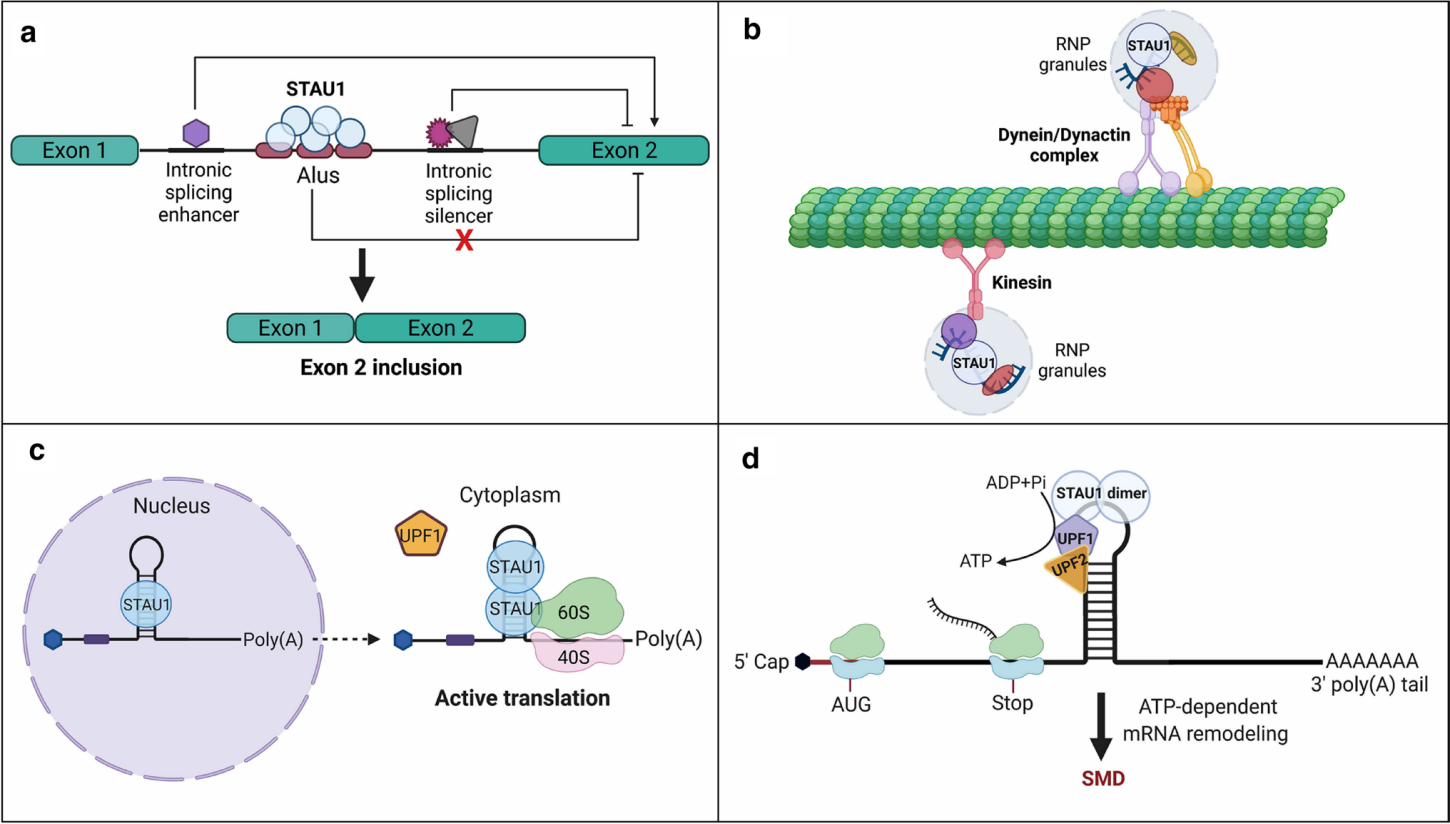
STAU1 binding to target mRNAs regulates various aspects of RNA metabolism. **a** STAU1 binding to SBS (e.g., Alu repeats) located in target mRNAs regulates alternative splicing events. **b** STAU1 direct interaction with cis-acting motifs or localization signals in 3’UTR of target mRNAs recruits motor proteins (i.e., dynein and kinesin) for active transport of mRNAs to distinct subcellular locations using cytoskeletal networks. **c** Simultaneous binding of STAU1 to mRNAs and ribosome induces mRNA translation. **d** STAU1-mediated mRNA decay (SMD) involves direct binding of STAU1 to SBS located downstream of the stop codon (3′UTR) of target mRNAs and recruitment of UPF1 and UPF2 helicases leading to mRNA degradation (Figure is created with BioRender.com)

In this context, STAU1 has been shown to play critical roles in multiple steps of RNA production including splicing, localization, stability, translation, and decay (Fig. 2) [1, 9, 15, 38]. For instance, the regulatory role of STAU1 in pre-mRNA splicing has recently been characterized in different studies (Fig. 2a) [9, 10, 36]. In particular, mass spectrometry analysis of STAU1-RNP complexes from Hela cells showed colocalization of STAU1 with splicing factors (SFRS13A, SFRS4, and SFPQ), thereby suggesting its role in splicing events [43]. Moreover, work from our lab has shown the key role of STAU1 in regulating splicing events in skeletal muscle cells in culture and in vivo [10, 34]. Furthermore, during the spatiotemporal localization of mRNAs, STAU1 plays essential roles in mRNA transport to different subcellular compartments [44]. These effects are dependent on the direct interaction of STAU1 with cytoskeletal and motor proteins. In this function, STAU1 first recognizes and interacts with cis-acting motifs or localization signals in 3ʹUTR of target mRNAs. Next, different factors including motor proteins (i.e., dynein and kinesin) are recruited to the site and actively transport mRNAs to distinct subcellular locations using cytoskeletal networks (Fig. 2b). The presence of dynein intermediate chain and kinesin heavy chain in STAU1-containing RNP complexes in mammalian cells supports the intermediate role of STAU1 in linking motor molecules and mRNA cargos [49]. For example, in human neural cells, STAU1-mediated transport of specific mRNAs via microtubules is essential for dendrite formation and morphological changes [45].

In addition to the above functions in splicing and transport, simultaneous interaction of STAU1 with actively translating ribosomes and 5′UTR of target mRNAs highlights its key role in mRNA translation (Fig. 2c). STAU1 localization with the rough endoplasmic reticulum (RER) emphasizes its role in transporting mRNA to the site of translation [14]. In mammalian cells, STAU1-mediated activation of mRNA translation requires the presence of a 5′ UTR SBS in target mRNAs. Results obtained from reporter assays using rabbit reticulocyte lysates and mammalian cultured cells expressing human immunodeficiency virus type 1 trans-activating response (TAR) element fused to the 5′ UTR of a reporter transcript, suggest that STAU1 binding to the 5’UTR of target mRNAs increases their translation [13].

STAU1 also plays critical roles in the regulation of mRNA stability, a fundamental control step in the rates of mRNA degradation in response to cellular environment. This process involves direct binding of stabilizing RBPs to the regulatory motifs of target mRNAs that promotes mRNA stability and enhances mRNA translation [11]. For example, in undifferentiated C2C12 myoblasts, direct binding of STAU1 to 3ʹUTR of Dvl2 mRNA enhances its stability and promotes cell proliferation. On the other hand, during myogenesis, a gradual reduction in STAU1 protein levels is accompanied by a reduced half-life of Dvl2 mRNAs and induction of myogenic differentiation [46]. In this context, STAU1-mediated mRNA decay (SMD) is an mRNA degradation process that involves direct binding of STAU1 to SBS located downstream of the stop codon (3′UTR) of the target mRNA [15]. Following recognition and binding of STAU1 to the dsRNA structures within the 3′UTR region of the target mRNA, a direct interaction of STAU1 with the ATP-dependent RNA helicase UPF1 enhances its helicase activity and promotes SMD [17]. Previous work showed that a competition between nonsense-mediated mRNA decay (NMD) and SMD is controlled by the preferential recruitment of the ATP-dependent RNA helicases UPF1 and UPF2 to the target mRNA [47]. However, a recent study suggested that STAU1 binding to UPF2 is more stable than its interaction with UPF1. Therefore, STAU1/UPF2 complex is likely responsible for recruiting UPF1 to the site of SMD [48] (Fig. 2d). SMD has been reported to play roles in various cellular processes including for instance, in the tight control of SMD-induced degradation of ADP-ribosylation factor 1 (Arf1) mRNA which is critical for regulating membrane traffic and organelle structure [49].

## Role of STAU1 in the regulation of cell functions and its associated impact on cancer

Given the significance of RBPs in the control of mRNA metabolism, their importance in cell fate have recently gained increasing attention. Therefore, over the past few years, a growing body of literature has demonstrated the role of a wide array of RBPs in the control of cell functions including growth, apoptosis, differentiation, and migration [50]. Importantly, due to the critical role of RBPs in the tight control of cellular functions, dysregulation in the expression pattern or function of these proteins contributes to the pathology of various diseases including cancer [51]. As discussed, the significant contribution of STAU1 in RNA metabolism affects the regulation of a wide variety of key cellular functions thereby leading many laboratories to focus their efforts on the role of STAU1 in the pathophysiology of various diseases [3, 30–32]. In this context, the significance of STAU1 in the pathogenesis of cancer has been reported to be linked to its role in the regulation of mRNA translation, splicing, and decay [32]. Table 1 shows the expression profile of STAU1 and its function in different cancers based on its role in RNA metabolism. In this section, we focus on the molecular mechanisms underlying STAU1-mediated control of cell functions and highlight its plausible effects on human cancer.

**Table 1:** Summary of STAUl expression profile and function in various cancers

Cancer	Cell line	STAUl expression	Cell function	mRNA metabolism	Observation	Impact
Colorectal cancer	HCT116, LS174T, HT-29	N/A	Cell cycle control, proliferation, apoptosis	Ribosome assembly, protein translation, SMD	Modulation of STAU1 expression impacts cell survival [18, 98]	Tumor promoting or tumor inhibiting
Osteosarcoma	U2OS	N/A	Cell cycle control and proliferation	N/D	An ectopic expression of STAUl impairs proliferation [18]	N/D
Glioblastoma	U87, U251	N/A	Tumor growth and metastasis	SMD	Increased SMD of RAX2 inhibits glioblastoma progression [75]	Tumor inhibiting
Gastric cancer	SGC7901, BGC823	N/A	Cell proliferation, in vitro migration and invasion	SMD	STAUl silencing reduces cell prolifera tion [12]	Tumor promoting
Embryonal rhabdomyosarcoma	RD	Increased (> 2.5-fold)	Cell proliferation, in vitro migration and invasion, tumorigenesis	Protein translation	STAUl silencing reduces ERMS progression [32]	Tumor promoting
Alveolar rhabdomyosarcoma	RH30, RH41	Increased (~ twofold)	Apoptosis, in vitro migration and invasion, tumorigenesis	Autophagy	STAUl silencing reduces ARMS progression via autophagy inhibition [32, 33]	Tumor promoting
Prostate cancer	LNCaP, DU145, PC3	Increased (> 2000-fold)	Cell proliferation, in vitro migration and invasion	N/D	STAU1 silencing reduces prolifera tion or metastasis of prostate cancer cells [31]	Tumor promoting
Neuroblastoma	SH-SY5Y, neuro-2a	N/A	Apoptosis, Neural growth, differentiation, dendrite formation and morphology,	mRNA localization, alternative splicing, SMD	STAUl silencing partially blocks neural cell differentiation and promotes apoptosis [28, 69, 86]	Tumor promoting or tumor inhibiting
Cervical cancer	HeIa	N/A	Cell migration	SMD	STAU1 depletion promotes cell migration in wound healing assay [91]	Tumor inhibiting

*N/A* Not Available, *N/D* Not Determined

### STAU1 and cell polarity

Polarity is a characteristic of eukaryotic cells that refers to a spatial organization of intracellular compartments with distinct organelles and proteins. Asymmetric localization of intracellular compartments allows simultaneous occurrence of several vital reactions in distinct subcellular domains. Cell polarity is essential for the asymmetric segregation of cell fate determinants during cell division [52]. For efficient preferential distribution of proteins and prevention of inappropriate protein translation in various cytoplasmic domains, intracellular localization of mRNAs prior to translation is a pivotal step which is achieved through the activity of a group of RBPs [53].

The significance of Staufen protein in cell polarity was initially described in Drosophila oocyte where Staufen binding to several maternal mRNAs promotes their anterior–posterior localization and regulates oocyte polarity. More specifically, during Drosophila development, *Oskar* is responsible for assembly of germ cell cytoplasm and formation of the posterior pole. *Oskar* and two other downstream mRNAs, *Vasa* and *Tudor* will be localized in polar granules where Staufen binding to *Oskar* facilitates granule localization at the posterior pole [1]. This step is key to early sex determination and maturation of the sex organs in Drosophila embryo. Similarly, microtubule-dependent mechanism of *Bicoid* mRNA transport to the anterior pole of Drosophila oocyte by Staufen is essential for oocyte polarity and maturation [54]. This effect is not limited to germ cells since Staufen-mediated asymmetric cortical localization of prospero mRNAs in mitotic neuroblasts regulates development and specification of ganglion mother cells. This step is essential for development of the Drosophila nervous system and for determination of mother-daughter cell fate [7]. In the absence of Staufen, Drosophila embryos show multiple defects in the anterior head, abdominal segments, and germ cell formation [1], while also exhibiting disruption in the long-term memory formation [55].

Similar observations have been made in other organisms. For instance, in *Xenopus oocytes*, Staufen localization in a ribonucleoprotein complex containing kinesin motor protein and the maternal mRNAs Vg1 and VegT plays a crucial role in targeting maternal mRNAs to the vegetal pole that is required for endoderm and mesoderm specification. Therefore, ectopic expression of mutant Staufen in *Xenopus oocyte* blocks the vegetal localization of Vg1 mRNA [56]. Furthermore, Staufen’s association with kinesin protein is required for oocyte maturation in pigs. Kinesin is a motor protein that actively moves along microtubule filaments through its ATP hydrolysis activity. It acts in a wide range of cellular functions such as cell division and intracellular transport of RNAs via the microtubule network. Also, the association of Staufen with kinesin-positive ribonucleoprotein complexes has been reported by several studies. Specifically, during maturation of pig oocytes, localization of mRNAs into the specific parts of the cytoplasm is driven by kinesin KIF5B that requires association of Staufen protein [57]. More recently, in zebrafish, STAU1 and STAU2 were shown to be essential for the primordial germ cell migration. Therefore, depletion of STAU1 or STAU2 led to the aberrant migration of germ cells and death of the embryo. These effects were linked to the aberrant expression of primordial germ cell-specific gene *vasa* in Staufen-depleted embryos. Asymmetric localization of vasa transcripts in the germplasm is critical for the specification of these cells that controls by the direct binding of STAU protein to the vasa mRNA [22].

Given that the maintenance of cell polarity is crucial for the proper functioning of organs, its disturbance is associated with various human diseases [58]. For example, the apical-basolateral polarity of the plasma membrane in intestinal epithelial cells is essential for the transport and uptake of nutrients [59]. The proper localization of distinct proteins and lipids in each domain occurs via cytoskeleton-dependent mechanisms that could involve STAU1. It has been shown that asymmetric distribution of STAU1 in the apical pole in the differentiated human intestinal epithelial cells Caco-2 is required for a preferential RNA localization in this site [20]. Similar examples emphasize the importance of STAU1-induced cell polarity in normal tissue development and functioning, highlighting the detrimental impact of STAU1 dysregulation on cells and organs [56, 60].

Disrupted cell polarity is one of the hallmarks of cancer [61]. Therefore, several cell polarity proteins are classified as tumor suppressors or proto-oncogenes [62]. While asymmetric cell division is a critical phenomenon for cell development and differentiation, aberrant cell polarity leads to symmetric cell division and promotes cancer cell growth [63]. More specifically, during asymmetric division of stem cells, a single cell generates two daughter cells with distinct fates: one retains the characteristics of the stem cell and divides asymmetrically to generate two distinct daughter cells. While the other one loses stem cell properties, differentiates, and specializes to the specific cell type which plays certain tasks in the body [64]. In cancer, the population of stem cells is being maintained via asymmetric cell division. However, disruption in the signaling pathways governing cell polarity and asymmetric cell division leads to the absolute production of stem-like cells through symmetric cell division. These cells carry the property to proliferate and further accumulate stem-like cancer cells with limited capacity to differentiate. As a result, a large population of poorly differentiated cancer cells will be generated which exhibit high metastatic potential [65].

Given the importance of STAU1 in developing and maintaining cell polarity, its dysregulation may affect cell polarity and impair asymmetric cell division thereby promoting cancer development and progression. Also, based on the available evidence on the crucial impact of STAU1 on both cell polarity and differentiation, STAU1 may play important roles in maintaining a balance between pluripotency and differentiation properties of stem-like cancer cells. In this context, STAU1 may exhibit tumor suppressor or oncogenic effect depending on the source and type of cancer stem cells as well as the type of tissues to which stem cells are destined to differentiate. For instance, the negative impact of STAU1 expression levels on the differentiation of mouse myoblasts (C2C12 cells) has been previously demonstrated [66]. Therefore, STAU1 level may inhibit myogenesis in cancer stem cells, and hence, promote muscle-related cancers. However, further studies may focus on detecting the direct interaction between STAU1 and the mRNAs of the apical–basal polarity markers (e.g., Crumbs3, Pals1, and Pals1-associated tight junction protein, Patj) in cancer cells [62]. In addition, the indirect impact of STAU1 on the control of cell polarity should be investigated separately. For instances, in cancer cells, loss of the apical-basal polarity is associated with acquiring the migratory phenotype that involves Epithelial–Mesenchymal Transition (EMT) process [67]. During EMT, transformed cells lose their polarity and cell–cell adhesion properties while expressing mesenchymal and pro-migratory genes. This transition is regulated by various signaling pathways including transforming growth factor β (TGF-β), Wnt, Notch, and Hedgehog pathways [68]. Accordingly, the role of STAU1 in these signaling pathways may indirectly impact cell polarity and cancer development.

### STAU1 and cell growth

In addition to cell polarity, STAU1 has been shown to be involved in cell growth by regulating mRNA translation of key components of the cell cycle. A recent study by Ghram et al. showed that STAU1-mediated post-transcriptional regulation of cell cycle mRNAs is essential for proliferation of non-transformed cells [19]. The expression level of over 30 cell cycle regulator transcripts was shown to be dysregulated in STAU1-depleted cells, suggesting the essential role of STAU1 in cell cycle control. This study also reported that STAU1 direct binding to the 3’UTR of E2F1 (an essential transcription factor in G1/S transition) mRNA contributes to its translation and promotes G1/S transition [19]. Furthermore, a recent study provided evidence on the critical role of STAU1 in neural cell survival and growth through regulation of alternative splicing and expression of genes (e.g., PDGFB, C–C motif chemokine ligand 2 or CCL2, OASL) its involvement in the nerve growth factor signaling pathway. Therefore, STAU1 expression appears critical for the proper splicing and expression of neural genes [69].

Other studies presented evidence on the impact of STAU1 on cell growth and proliferation in various in vitro [70–72] and in vivo models [73]. These effects can result from positive or/and negative control of gene expression by STAU1 leading to the inhibition or activation of cell growth. The critical importance of cell growth control mechanisms in cancer progression [74] accompanied by the observed roles of STAU1 in cell growth, led to recent investigations on the impact of STAU1 on cell growth linked to the pathophysiology and treatment of various cancers [18, 32, 33]. Examination of the STAU1 levels in proliferating human transformed cell lines HCT116 (colon cancer cells) and U2OS (osteosarcoma cells) at different stages of the cell cycle, indicated that STAU1 protein levels increase during the early phases of the cell cycle (S and G2 phases) and rapidly drops later in mitosis. While STAU1 mRNA levels remained unchanged, STAU1 proteins were degraded by the anaphase-promoting complex (APC) ubiquitin–proteasome system as cells enter mitosis. In particular, the interaction between STAU1 protein and the APC/C adapter proteins Cdc20 and Cdh1 promotes its proteasomal degradation in mitotic cells. Therefore, ectopic expression of STAU1 in these cells impaired proliferation, showing that the tight regulation of STAU1 levels is necessary to prevent the detrimental impacts of STAU1 on mitosis [18]. Moreover, in HCT116 cells, STAU1 has been shown to affect cell growth by controlling localization of a group of pre-rRNAs during mitosis. This may play critical roles in the assembly of ribosomes and protein translation [8].

STAU1-mediated mRNA decay has been shown to also play a significant role in the control of cancer cell proliferation [75, 76]. A recent study in glioblastoma indicated that SMD of the transcription factor retina and anterior neural fold homobox2 (RAX2) transcript is required for inhibition of tumor growth and metastasis. These findings show that STAU1 interaction with a ribonucleoprotein complex containing RAX2 transcripts, brain-derived neurotrophic factor antisense (BDNF-AS) lncRNA, and RNA helicase UPF1 promotes SMD of RAX2 mRNAs and inhibits glioblastoma progression [75]. On the other hand, a recent study showed that increased STAU1-mediated degradation of zinc-finger protein 331 (ZNF331) (a transcription suppressor which plays tumor suppressor function in different cancers including gastric and colorectal cancers) mRNAs is associated with the growth of glioma cells (U87 and U251) and directly correlates with tumor grades (stages III and IV). Hence, STAU1 depletion increased stability and half-life of ZNF331 mRNAs and inhibited glioma progression [76]. Based on these findings, STAU1 may thus play differential roles in in vitro models versus tumors tissues of brain cancers based on the disease stage and its direct mRNA targets. Accordingly, proper regulation of SMD may exert anti-tumor effects in high-grade gliomas. Similarly, the available survival data for glioma patients show a negative relationship between STAU1 mRNA levels and the 3 year survival rate, supporting its oncogenic role in gliomas [77]. Furthermore, as suggested, STAU1 expression levels can also be considered as a potential biomarker for high grade gliomas [76].

In gastric cancer cell lines (SGC7901 and BGC823), upregulated TINCR lncRNA forms a ribonucleoprotein complex with STAU1, UPF1, and KLF2 mRNA that promotes SMD of KLF2 transcripts. Reduced levels of the transcription factor KLF2 decreases mRNA expression of two important target genes, cyclin-dependent kinase CDKN1A/P21 and CDKN2B/P15 (inhibitors of cell cycle checkpoints and cell proliferation) that promote gastric cancer cell growth. In addition, knockdown of STAU1 or overexpression of KLF2 in gastric cancer cells increased expression levels of cyclin-dependent kinases and reduced cell proliferation [12]. An independent study supported these findings by showing that STAU1-mediated degradation of p21 in HOXA11-AS-overexpressing gastric cancer cells (BGC823 and SGC7901 cell lines) promotes proliferation. Inhibiting p21 mRNA degradation by SMD leads to cell cycle arrest and reduces growth of gastric cancer cells[78]. These findings suggest an oncogenic function of STAU1 in gastric cancer cell lines linked to its role in SMD. Therefore, tight regulation of SMD in this cancer type may improve its treatment and prognosis.

STAU1 can also regulate cancer cell survival through translational regulation. We have recently reported that in embryonal rhabdomyosarcoma cells (RD), elevated expression of STAU1 is associated with increased cell proliferation. Hence, genetic silencing of STAU1 in vitro reduces growth of cancer cells and inhibits tumor formation in vivo. The observed pro-growth effect of STAU1 on rhabdomyosarcoma cells was due, in part, to the increased translation of oncogenic c-myc via direct binding of STAU1 to its mRNA [32]. Similar observations have been reported in prostate cancer cell line LNCaP, indicating that STAU1 downregulation inhibits proliferation of these cells without promoting apoptosis [31]. Along those lines, STAU1 has been previously shown to be an unfavorable prognostic marker in prostate cancer [77]. These findings provide evidence on the positive impact of STAU1 on cancer cell growth and shed light on the plausible therapeutic potential of STAU1 targeting for cancer treatment.

### STAU1 and cellular differentiation

Cellular differentiation is a multistep process that generates a specialized cell type from a primary cell. Differentiation is one of the main events during development of multicellular organisms that leads to generation of different organs with distinct characteristics and functions. During differentiation, dividing cells withdraw from the cell cycle and begin to express a group of genes required for the specific function of the differentiated tissue [79].

Given the significant role of RBPs in the spatio-temporal regulation of gene expression in response to various cellular events including differentiation, the role of STAU1 in cellular differentiation has been broadly studied. As a result, the impact of STAU1 on epidermogenesis [80], myogenesis [66, 81], neurogenesis [82], and adipogenesis [21, 83] has been well established. During epidermal differentiation, STAU1 functions to stabilize mRNAs of key differentiation factors. More specifically, TINCR (terminal differentiation-induced ncRNA) is required to guide STAU1 protein towards the target mRNAs. Therefore, the TINCR-STAU1 complex is necessary for the abundance of differentiation mRNAs including KRT80, ALOXE3, ALOX12B, ELOVL3, and FLG. While mutation in any of these genes causes different skin disorders in humans, depletion of STAU1 or TINCR genes disrupts epidermal terminal differentiation [80]. By contrast, in skeletal muscle, STAU1 negatively affects myoblast differentiation by regulating the stability and translation of the myogenic mRNAs [9, 66]. It has been reported that STAU1 depletion in C2C12 myoblasts increased expression of myoglobin (a muscle-specific iron- and oxygen-binding protein) and myogenin (a muscle-specific transcription factor involves in skeletal muscle development) while promoting spontaneous activation of myogenesis [81]. Similarly, we reported that STAU1 overexpression in C2C12 prevents myogenic differentiation by reducing the expression of MyoD, myogenin, MEF2A, and MEF2C via SMD-independent mechanisms [66]. Moreover, GO enrichment and KEGG pathway analysis revealed that STAU1 regulates alternative mRNA splicing of genes involved in muscle cell differentiation [69].

Further studies showed that in cultured hippocampal neurons derived from STAU1 mutant mice (homozygous STAU1^tm1Apa^ mouse expressing defective STAU1 protein which lacks RNA binding ability), the density of dendrites and synapses were reduced. These effects were accompanied by the aberrant delivery of STAU1-containing ribonucleoprotein vesicles to dendrites of hippocampal neurons and, therefore, a reduced locomotor activity in the STAU1 mutant mice [82]. Moreover, during adipogenesis, STAU1 direct binding to the 3′UTR of Kruppel-like factor 2 (Klf2; an anti-adipogenic factor) mRNA promotes its degradation and facilitates adipocyte differentiation. In this context, downregulation of SMD components including STAU1, inhibited mouse adipogenesis which was restored by exogenous expression of Klf2 [21]. Similarly, suppressor of morphogenesis in genitalia 1 (SMG1) was shown to promote adipogenesis via facilitating STAU1 and UPF1 colocalization leading to SMD activation [83]. Altogether, these findings reveal the critical role of STAU1 in cell differentiation.

Given the fact that most cancers exhibit poorly differentiated or undifferentiated cellular phenotypes, the importance of differentiation process in cancer development is evident. It is well-established that the differentiation stage of a tumor is linked to tumor behavior and aggressiveness. Therefore, in aggressive types of cancer, cells mostly undergo proliferation and dedifferentiation [9]. It has been shown that poorly differentiated cancers have enhanced ability to invade through the deeper layers of the dermis and metastasize to lymph nodes, leading to poor patient prognosis [84]. As discussed above, given the multifunctional nature of STAU1 in controlling cellular differentiation [85], STAU1 dysregulation may positively or negatively impact cancer cell differentiation and contribute to the severity of the disease.

In this context, the role of STAU1 in differentiation of the human neuroblastoma cell line SH-SY5Y and dendritic development has been studied by various groups [45, 86, 87]. It has been shown that in differentiated SH-SY5Y cells, localization of ribonucleoprotein complexes containing STAU1 in soma and dendrites is essential for appropriate dendrite formation and morphology. The association of ribosomal components with STAU1-positive granules in dendrites highlights STAU1’s function in the translational machinery and its role in the translation of required proteins for dendritic development. In addition, the association of STAU1 with the cytoskeleton and motor proteins such as *β*-actin, *α*-tubulin, kinesin, dynein, FMRP, and Tau, suggests STAU1’s involvement in mRNA transport to the specific sites of the cell during differentiation [45]. A separate study revealed that elevated expression of STAU1 during differentiation of SH-SY5Y cells is required for proper development of dendrites. Moreover, in SH-SY5Y cells, the abundance of the brain-specific microRNA miR-124 (an important player in neuronal differentiation [87]) in STAU1-positive vesicles emphasizes the significance of STAU1 in neuroblast differentiation. Along those lines, siRNA-mediated silencing of STAU1 in SH-SY5Y cells partially blocks cell differentiation and alters dendrite organization, density, and length [86]. These findings reveal the positive role of STAU1 in the differentiation of neuroblastoma cells that may further impact the stage of the disease. Moreover, in Hela and Neuro-2a cell lines, STAU1 has been shown to regulate alternative splicing of genes involved in neural growth and differentiation such as PLEKHG2 and ARHGEF1 [69]. Thus, proper regulation of STAU1-mediated alternative splicing in these cells is crucial for normal neural growth, proliferation, and axon development [69].

As discussed earlier, polarity and asymmetric division are the main events contributing to proper cellular differentiation. Given the essential roles of STAU1 in these processes and the importance of cell differentiation in cancer development, STAU1 dysregulation may further impact cancer progression through controlling cellular differentiation. Therefore, the direct or/and indirect impact of STAU1 on cell polarity, EMT, and cell–cell adhesion may contribute to the poor-differentiation phenotype of cancer cells and disease severity.

### STAU1 and cell migration

Cell migration is a natural process during embryonic development, wound healing, and immune response. Tight regulation of cell movement is critical for the proper development of organisms and response to stimuli [88]. Accordingly, uncontrolled cell migration can cause various developmental problems and promote tumor metastasis [89]. In this context, STAU1 has been reported to be required for the proper migration of primordial germ cells (PGCs) during gametogenesis in zebrafish. Therefore, in embryos lacking STAU1, expression of the PGC marker, vasa, was reduced and PGC migration was aberrant. Importantly, mis-migrating PGCs failed to survive in the STAU1-compromised embryo, highlighting the significance of STAU1 in germline and embryo development by regulating cell migration [22]. More recently, STAU1 has been reported to regulate alternative splicing and expression level of CCL2 mRNAs whose protein product is responsible for leukocyte migration and inflammatory response [69]. Since cell migration is a critical step in tumor progression [89], several studies uncovered role of STAU1 in the regulation of cell migration during cancer development and tumorigenesis. In this regard, STAU1 has been shown to regulate cancer cell migration, positively or negatively, through different mechanisms. Therefore STAU1 has been suggested as a novel therapeutic target for the inhibition of cancer metastasis [31, 32, 90, 91].

STAU1 is known to negatively control migration of Hela cells by promoting mRNA degradation of SERPINE1 and RAB11-family-interacting protein 1 (RAB11FIP1) through SMD. Moreover, STAU1 depletion in Hela cells increased SERPINE1 and RAB11FIP1 expression and promoted cell migration in wound healing assay [91]. A recent study on glioma cell lines (U251 and U87) further demonstrated that STAU1 blocks cell migration and invasion by degrading metal regulatory transcription factor 1 (MTF1) and YY2 transcription factor (YY2) through SMD [90]. Similar observations have been made in U251 and U87 cell lines, where overexpression of lncRNA brain-derived neurotrophic factor antisense (BDNF-AS) reduced cell migration by promoting SMD of the retina and anterior neural fold homeobox 2 (RAX2) mRNA [75].

In rhabdomyosarcoma cell lines (RH30 and RD), however, elevated STAU1 promotes cell migration and invasion. Therefore, genetic silencing of STAU1 in these cells reduced cell metastasis and inhibited cancer progression [32]. A similar effect was observed in gastric cancer cells and tumors in which STAU1-mediated degradation of the KLF2 transcription factor, promotes in vitro and in vivo metastasis. Therefore, inhibition of SMD restored KLF2 mRNA expression and reduced cell migration and invasion [78]. In the prostate cancer cell lines PC3 and DU145, elevated STAU1 was associated with increased migration and invasion through FAK signaling. More specifically, the presence of STAU1-binding sites in the mRNAs of several integrins (the upstream regulators of FAK pathway), SHP-2 (a phosphatase dephosphorylating FAK), and Scr (a kinase responsible for FAK phosphorylation) suggests a direct and multifunctional role of STAU1 in controlling FAK signaling [31]. Therefore, STAU1 downregulation partially inhibited the motility and metastasis of prostate cancer cells, suggesting that STAU1 targeting may exhibit a therapeutic effect for prostate cancer treatment [31]. Taken together with other data showing a correlation between STAU1 levels and advanced stages of cancer, these findings suggest a promising cancer-specific role for STAU1 in the regulation of tumor metastasis.

### STAU1 and cell death

Cell death can naturally occur in old cells to replace them with new and healthy ones. However, aging, different diseases, and injuries can also promote programmed cell death or apoptosis [92]. Although apoptosis is crucial for normal body functions, embryonic development, proper functioning of the immune system, and response to fatal stimuli, inappropriate apoptosis causes several health complications such as autoimmune diseases, neurodegeneration, and cancer [93]. This explains why apoptotic pathways are tightly regulated by intracellular control mechanisms [94, 95].

In the past two decades, the role of several RBPs has been investigated in the activation and/or inhibition of apoptosis [96]. For instance, direct binding of HuR to the 3′UTR of apoptotic protease activating factor 1 (Apaf-1) mRNAs promotes their stability and activates caspase-dependent apoptosis of carcinoma cell lines [97]. Similarly, STAU1 has been linked to the regulation of apoptosis in both non-transformed and malignant cells [23, 32]. A recent study demonstrated that STAU1 expression is required for apoptosis induction in response to Unfolded Protein Response (UPR) in neural cells. This study reported that in response to ER stress, the elevated level of STAU1 in neural cells promotes apoptosis by activating PERK-CHOP pathway and leads to neurodegeneration [23]. Therefore, neural cells derived from STAU1^−/−^ mice showed reduced UPR activity and apoptosis in response to thapsigargin-induced cellular stress. Based on this, STAU1 depletion has been proposed as a therapeutic approach in spinocerebellar ataxia type 2 (SCA2) mouse model [23, 27].

The pro-apoptotic function of STAU1 has been also observed in cancer. More specifically, in the colorectal cancer cell lines HCT116, LS174T, and HT-29, SMD inhibition by long non-coding RNA SNHG5 promotes cancer cell survival via increasing the stability of several SMD target mRNAs including Spermatogenesis Associated Serin Rich 2 (SPATS2). Therefore, SMD-mediated degradation of SPATS2 transcripts promotes apoptosis and inhibits cancer progression. By contrast STAU1 depletion blocks apoptosis and increases survival of colorectal cancer cells [98]. Several studies also emphasized the anti-apoptotic role of STAU1 via the control of mRNA stability and translation in neuroblastoma cells. In SH-SY5Y cell line, a complex of TDP-43/FMRP/STAU1 proteins binds to the 3’UTR of Sirtuin1 (SIRT1) mRNA and promotes its stability and translation. STAU1 downregulation reduced both mRNA and protein levels of SIRT1 which leads to apoptosis of SH-SY5Y cells [28]. Given the critical role of SIRT1 in the NAD^+^ pathway and its importance in the control of cell differentiation, apoptosis, autophagy, metabolism, and stress response, STAU1 may indirectly regulate fate of neuroblastoma cells through controlling SIRT1 levels. Likewise, in the alveolar rhabdomyosarcoma cell line RH30, STAU1 knockdown causes apoptosis and reduces cancer progression [32]. Altogether, these studies provide solid evidence on the role of STAU1 in the control of apoptosis that may impact tumorigenesis. However, the apparent distinct roles of STAU1 in controlling apoptosis among various cancer types may ultimately determine its differential impact as either an oncogene or tumor suppressor. Given this, the antitumor therapeutic effect of STAU1 should be investigated separately in each cancer type and at different stages of the disease.

### STAU1 and autophagy

Macroautophagy or autophagy is a natural intracellular degradation process that involves the autophagic-lysosomal degradation of intracellular compartments and protein aggregates. During cellular stress, autophagy activation determines cell fate by controlling a balance between cell survival and cell death [99]. The essential role of autophagy in maintaining intracellular homeostasis [100] has led researchers to further investigate its impact on cell integrity and function as well as its contribution to various diseases including cancer [101]. Autophagy has been shown to be regulated by a group of RNA-binding proteins such as Human antigen R (HuR) [102] and Zinc Finger 423 (ZNF423) [103]. The impact of STAU1 in autophagy control has only recently emerged but the limited available evidence supports an important role for STAU1 on autophagy control in non-transformed and malignant cells.

Recently, Paul et al. showed that autophagy is involved in the degradation of STAU1 protein in the in vitro models of SCA2 and amyotrophic lateral sclerosis (ALS) [27]. Therefore, aberrant autophagy leads to STAU1 accumulation and dysregulates its function. A separate study conducted by the same group indicated that STAU1 protein controls autophagy in HEK293 cells via direct binding to the 3′UTR region of mTOR mRNA and promotion of its translation. Therefore, STAU1 expression led to upregulation of the mTOR signaling pathway and autophagy inhibition [24]. A recent study from our lab supports these findings by showing that STAU1 silencing in normal mouse myoblasts (C2C12) promotes autophagy via reducing mTOR protein levels. Moreover, exogenous expression of STAU1 in C2C12 myoblasts and human skeletal muscle cell lines (HSMM-C2 and HSMM-C3) inhibits autophagy in a mTOR-dependent manner [33]. Altogether, these findings revealed that STAU1 regulates autophagy degradation in mammalian cells.

In cancer, our recent findings indicated that STAU1 positively impacts autophagy in the alveolar rhabdomyosarcoma cell lines RH30 and RH41 through upregulation of JNK signaling pathway or direct interaction with the mRNA of autophagy-related genes (ATGs). Therefore, STAU1 depletion led to autophagy inhibition and apoptosis induction [33]. Given the available evidence on the role STAU1 in the control of two main autophagy regulatory pathways, mTOR [24, 33] and JNK [33], as well as its direct binding to ATG mRNAs [33], further studies are necessary to determine the exact role of STAU1 in autophagy control of normal and cancer cells. In addition, due to the dual but contrasting effects of autophagy modulation in cancer [104], STAU1-mediated autophagy control may differentially impact cancer progression under various circumstances (i.e., tumor type, disease stage, treatment regimen, mutational status of tumor suppressors and oncogenes, etc.).

### STAU1 and response to stress (stress granules)

Stress granules (SGs) are cytosolic aggregates that contain proteins and RNAs. Under certain stressful circumstances, SGs form transiently in cells to stall mRNA translation. Therefore, a combination of polyadenylated mRNAs, poly-A binding protein (PABP), translation initiation factors, and a group of RBPs, accumulate in stress granules. Formation of these dynamic cytoplasmic structures has been proposed to play critical roles in mRNA fate by acting as a transition point between mRNA storage, translation, and degradation [105]. A growing body of evidence suggests that STAU1 is an essential component of SGs which regulates formation of SGs, thereby emphasizing STAU1’s role in cell survival under stress [25] and diseased conditions [26, 27]. In mammalian cell cultures, NIH 3T3, HeLa, and BHK cell lines, STAU1 impairs stress granule formation upon stress. However, STAU1 is required for stabilization of polysomes and recovery from stress [25]. Likewise, STAU1 downregulation in the in vitro model of Muscular Dystrophy Type1 (DM1) rescues stress granule formation in response to arsenite-induced stress. Therefore, the negative impact of STAU1 overexpression on stress granule formation in DM1 myoblasts impaired the stress response and exacerbated disease phenotype [26]. Furthermore, in SCA2, STAU1 was associated with mutant ATXN2 in stress granules. Therefore, STAU1 silencing reduced accumulation of mutant ATXN2 protein and ameliorated disease phenotype [27].

Although there is no evidence on the role of STAU1 in cancer development through control of SGs formation, the impact of SGs on tumorigenesis and chemotherapy has been previously reported [106]. Several cancer signaling pathways (e.g. mTOR and RAS) have been shown to promote SG formation to enhance cancer cell survival, especially under stress. Accordingly, increased SG formation in cancer has been shown to promote tumorigenesis [106]. Thus, SGs have been suggested as a new therapeutic target for cancer treatment [107]. Given the available evidence on the tumorigenic function of STAU1 in several cancers as well as its involvement in the regulation of cancer signaling pathways such as mTOR accompanied by its role in SG formation, STAU1’s impact on cancer progression via controlling SG formation clearly warrants further research. For instance, the direct role of STAU1 in upregulating the mTOR pathway may positively impact formation of SGs and further promote cancer cell growth and survival. In this case, STAU1 downregulation may inhibit cancer cell survival by preventing SG formation under stress. On the contrary, based on the available evidence on the negative impact of STAU1 levels on SG formation in myoblast [26], STAU1 may alternatively function as a tumor suppressor in some cancers by inhibiting SG formation. Given these findings, the context-specific role of STAU1 in SG formation needs to be studied carefully in different cancer types.

## Conclusion and perspective

Over the past two decades, the role of STAU1 in the regulation of cell functions has been broadly investigated. Studies conducted in non-transformed cells have demonstrated that STAU1 expression is required for the proper control of several cell functions such as cell polarity, cell cycle transition, and differentiation [19, 22, 57, 66]. These effects are associated with an alteration in the expression level of STAU1 at different stages of cell development with for example, STAU1 level fluctuating during different stages of cell cycle and cellular differentiation [19, 66]. These observations provide compelling evidence showing that a tight regulation of STAU1 levels in healthy cells is critical for the proper functioning of the organism. Therefore, dysregulated STAU1 expression may contribute to the pathophysiology of various diseases. In this context, the controversial role of STAU1 in regulating cell growth serves as a good example to highlight the differential impact of STAU1 levels in normal versus transformed cells. During normal cell growth, STAU1 expression is be essential for promoting G1/S transition and positively control of cell proliferation [19]. However, STAU1 upregulation in cancer cells causes increased growth which may lead to adverse prognosis in cancer. On the other hand, given the important role of STAU1 in cellular differentiation [21, 66, 80–83], upregulated STAU1 in cancer cells may promote cellular differentiation and positively affect cancer prognosis. Overall, depending on the dysregulated cell function, STAU1 levels may exert positive or negative impact on cellular health and disease progression.

**Fig. 3:**
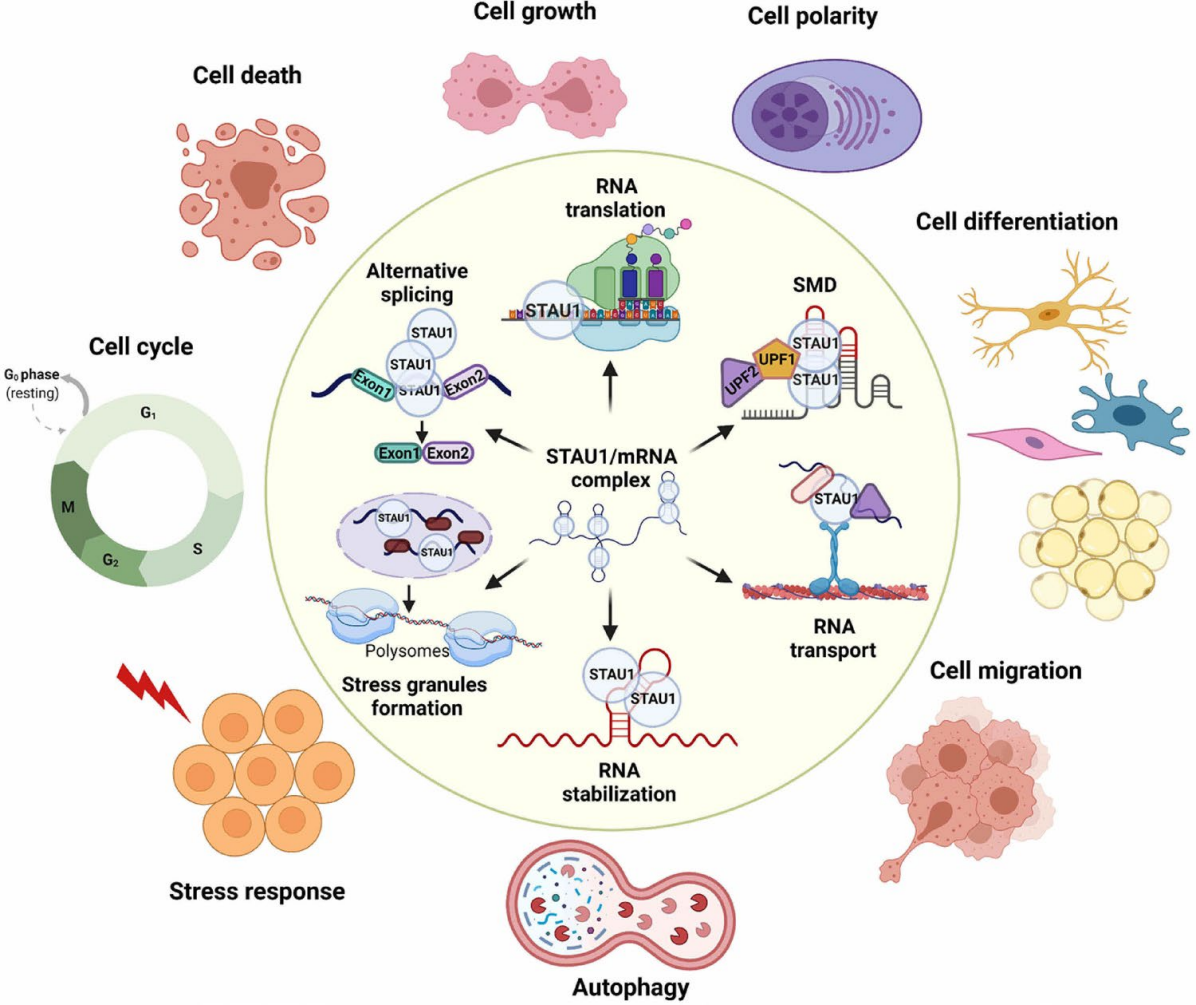
STAU1 direct binding to target mRNAs regulates different cellular functions via controlling mRNA metabolism. Formation of STAU1/RNA complex controls RNA translation, SMD, RNA transport, RNA stabilization, stress granule formation, and alternative splicing. Alterations in the metabolism of mRNAs markedly affect cellular functions including cell growth, polarity, differentiation, migration, autophagy, stress response, cell cycle control, and cell death. Modulation of each of these cell functions may promote oncogenesis (figure is created with BioRender.com)

These variations in STAU1 functions are not only limited to healthy versus diseased cells, but they are also seen in different types [31, 32, 108] and stages [76] of the same disease. For instance, the available evidence indicates that in several malignancies, upregulated STAU1 may act as an oncogene to manipulate normal cell functions and promote cancer development while in other cancers, STAU1 may act as a tumor suppressor and inhibit disease progression (Table 1). Also, STAU1 level was shown to be correlated with a specific stage of glioma [76]. The observed discrepancies in the role of STAU1 among different cancers and different stages can be due to the distinct regulatory effects of STAU1 on various cancerous signaling pathways. For instance, the critical roles of STAU1 in the regulation of mTOR, JNK, and FAK signaling might be key to its cancer type-specific function [24, 33]. However, given the heterogeneity and multifactorial nature of cancers, various underlying mechanisms may contribute to the differential functions of STAU1 among cancer types (Fig. 3) [31–33].

Based on these observations, STAU1 may thus serve as a potential therapeutic target for the development of novel cancer-specific treatments [31–33]. However, the cancer-specific effect of STAU1 targeting needs to be addressed when proposing it as an anti-cancer therapeutic target. Given the essential role of STAU1 in RNA metabolism and maintenance of the normal functioning of non-transformed cells, the anti-survival effect of STAU1 targeting on normal cells and tissues needs to be widely investigated to minimize its associated side effects. In this regard, a recent study has shown the importance of STAU1 in the proliferation of non-transformed human cell lines (hTERT-RPE1 and IMR90) [19], suggesting the plausible detrimental impact of STAU1 depletion in non-transformed cells. Despite the apoptotic impact of STAU1 depletion on malignant muscle cells, STAU1 depletion did not promote apoptosis in non-transformed myocytes [33]. The observed controversial effects of STAU1 depletion on different types of non-transformed and cancer cells emphasize the fact that developing a STAU1-based anticancer therapy requires further investigation on the mechanistic roles of STAU1 in both non-transformed and cancer cells.

Moreover, recent studies suggested that STAU1 may also act as a molecular biomarker for cancer diagnosis and prognosis [31, 32]. For instance, in prostate cancer, STAU1 has been reported as an unfavourable prognostic marker [31, 77]. Similarly, in glioma, head and neck, pancreatic, cervical, urothelial, thyroid, ovarian, and liver cancers, high level of STAU1 is correlated with poor patient survival [77]. In contrast, high level of STAU1 mRNA in lung, renal, and stomach cancer patients correlates with better patient survival [77]. Given the lack of relevant evidence, further investigation is required to identify the correlation between STAU1 levels and disease stage and severity. Overall, our knowledge of STAU1 involvement in cancer is still in its infancy. Accordingly, increased efforts should be invested in exploring the role of STAU1 in different cancers to reveal its full therapeutic potential.

## Data Availability

Not applicable.
